# Human Cytomegalovirus gH/gL Forms a Stable Complex with the Fusion Protein gB in Virions

**DOI:** 10.1371/journal.ppat.1005564

**Published:** 2016-04-15

**Authors:** Adam L. Vanarsdall, Paul W. Howard, Todd W. Wisner, David C. Johnson

**Affiliations:** Department of Molecular Microbiology and Immunology, Oregon Health & Science University, Portland, Oregon, United States of America; University of Alabama at Birmingham, UNITED STATES

## Abstract

Human cytomegalovirus (HCMV) is a ubiquitous virus that is a major pathogen in newborns and immunocompromised or immunosuppressed patients. HCMV infects a wide variety of cell types using distinct entry pathways that involve different forms of the gH/gL glycoprotein: gH/gL/gO and gH/gL/UL128-131 as well as the viral fusion glycoprotein, gB. However, the minimal or core fusion machinery (sufficient for cell-cell fusion) is just gH/gL and gB. Here, we demonstrate that HCMV gB and gH/gL form a stable complex early after their synthesis and in the absence of other viral proteins. gH/gL can interact with gB mutants that are unable to mediate cell-cell fusion. gB-gH/gL complexes included as much as 16–50% of the total gH/gL in HCMV virus particles. In contrast, only small amounts of gH/gL/gO and gH/gL/UL128-131 complexes were found associated with gB. All herpesviruses express gB and gH/gL molecules and most models describing herpesvirus entry suggest that gH/gL interacts with gB to mediate membrane fusion, although there is no direct evidence for this. For herpes simplex virus (HSV-1) it has been suggested that after receptor binding gH/gL binds to gB either just before, or coincident with membrane fusion. Therefore, our results have major implications for these models, demonstrating that HCMV gB and gH/gL forms stable gB-gH/gL complexes that are incorporated virions without receptor binding or membrane fusion. Moreover, our data is the best support to date for the proposal that gH/gL interacts with gB.

## Author Summary

Like all herpesviruses, HCMV expresses two envelope proteins, gH/gL and gB that are essential for entry. Models for how herpesvirus gB and gH/gL molecules function describe binding of gH/gL to gB that leads to conformational changes and activation of membrane fusion and virus entry. However, no evidence for direct binding of any gH/gL molecule to gB, especially from infected cells or virus particles, has been described. We report the novel observations that HCMV gB and gH/gL form stable, preformed complexes in extracellular virions independent of receptor binding. These observations are fundamentally important for understanding how herpesvirus glycoproteins mediate entry into cells. Moreover, the description of gB-gH/gL complexes in virions has major implications in terms of designing HCMV vaccines.

## Introduction

Entry of all herpesviruses into cells requires at least two membrane glycoproteins: gB and gH/gL that form the primordial and core fusion machinery. The structures of HSV-1 and Epstein Barr virus (EBV) gB molecules are similar to the vesicular stomatitis virus (VSV) G-protein, which is a type III fusion protein and strongly suggest that herpesvirus gB proteins are all fusion proteins [1,2]. However, unlike VSV G-protein, herpesvirus gB molecules require additional proteins for membrane fusion and entry. The most extensive data involves the α-herpesvirus HSV-1 that expresses glycoprotein gD that binds receptors, then it is believed that gD interacts with glycoprotein gH/gL that then interacts with gB to trigger membrane fusion [3]. For the γ-herpesvirus EBV, gH/gL or gH/gL/gp42 binds receptors and may transmit signals to gB for fusion [4]. Similarly, we have proposed that the β-herpesvirus human cytomegalovirus (HCMV) utilizes different forms of gH/gL, a trimer of gH/gL/gO and a pentamer of gH/gL/UL128-131 to bind distinct receptors and this promotes gB-mediated entry fusion [5]. Thus, a major tenet of all the models describing entry of α-, β-, and γ-herpesviruses suggests that gH/gL is activated by receptor binding (or by gD for HSV-1) and then binds to gB and in so doing, triggers gB for fusion.

At present, evidence for direct interactions between herpesvirus gB and gH/gL molecules has relied on indirect measurements involving transfected cells or *in vitro* studies. Bimolecular complementation (BiMC) studies involving HSV-1 gB and gH/gL fused to fragments of fluorescent proteins (YFP or GFP) and transfected into cells demonstrated that gB and gH/gL interact, but only after gD first interacts with receptors and then interacts with gH/gL [6,7]. HSV-1 gB and gH/gL interactions coincided with cell-cell fusion and HSV-1 gB “fusion loop” mutants that fail in fusion did not interact with gH/gL, suggesting that insertion of fusion loops into membranes coincides with or precedes the interaction [8]. However, the interpretation of the BiMC results was complicated by other observations that these HSV-1 glycoprotein-YFP fusion proteins interacted illegitimately with paramyxovirus glycoprotein-YFP constructs involving affinities between complementary YFP fragments [9]. Other important evidence for HSV-1 gB-gH/gL interactions involved *in vitro* experiments in which soluble gB associated with liposomes through “fusion loops” and promoted association of soluble gH/gL with these liposomes [10]. There was also evidence that HCMV gB and gH/gL can interact based on FRET analyses [11]. Many studies support the hypothesis that herpesvirus gH/gL proteins triggers gB glycoproteins for fusion, but there has been no direct evidence for this with wild type glycoproteins in virus-infected cells or in virus particles.

HCMV entry into fibroblasts involves fusion with the plasma membrane and requires gB and gH/gL/gO [12,13]. Entry into epithelial and endothelial cells involves macropinocytosis or endocytosis that requires gB, gH/gL/gO and gH/gL/UL128-131 [13–15]. We provided evidence involving interference studies that suggest gH/gL/gO binds critical receptors on fibroblasts and gH/gL/UL128-131 binds receptors on epithelial and endothelial cells [16,17]. Importantly, virus entry differs from HCMV glycoprotein-mediated cell-cell fusion, which requires only gB and gH/gL (without gO or UL128-131) and defines gB and gH/gL as the minimal fusion machinery [18].

In our previous studies of cell-cell fusion we observed that a HCMV gH/gL-specific antibody coprecipitated gB but these results were highly preliminary, no available gB antibodies precipitated gH/gL [18]. Here, we demonstrate that panels of gB-, gH- and gL-specific antibodies, as well as tagged gB, precipitated gB-gH/gL complexes from cells without the requirement of other HCMV proteins. Surprisingly, gB bound to gH/gL early after glycoprotein synthesis in the endoplasmic reticulum (ER). The interaction was observed in HCMV infected cells and in extracellular virus particles with 16–50% of the total gH/gL bound to gB. gH/gL/gO and gH/gL/UL128-131 bound poorly to gB in virions. Therefore, a significant fraction of HCMV gH/gL (devoid of gO or UL128-131) forms stable complexes with gB in virions and these interactions do not require receptors. Moreover, mutant forms of HCMV gB that lacked the capacity to mediate membrane fusion (e.g. fusion loop mutants) interacted with gH/gL so that fusion activity of gB is not necessary. These data provide a different model for how HCMV gB and gH/gL molecules interact and function compared with models for HSV-1 entry. Our data support the hypothesis that stable, preformed HCMV gB-gH/gL complexes are present in the virion envelope and that gB-gH/gL complexes might initiate membrane fusion between the virion envelope and cellular membranes.

## Results

### HCMV gB and gH/gL can be co-immunoprecipitated from Ad-transduced cells

**Fig 1:**
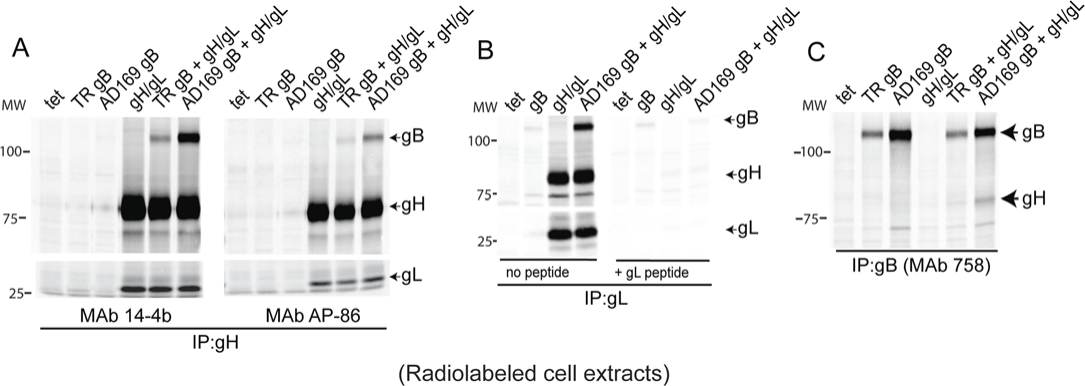
Detection of gB-gH/gL complexes following IP from radiolabeled Ad-transduced cells. ARPE-19 cells were transduced with Ad vectors expressing HCMV glycoproteins: HCMV strain TR or strain AD169 gB, TR gH/gL or a negative control, Ad-tet-trans (tet), as indicated at the top part of each panel. At 20 hrs post-transduction, the cells were radiolabeled with ^35^S-cysteine-methionine for 4 hrs. The cells were lysed with IP buffer containing 1% NP-40 and the proteins immunoprecipitated (IP’d) then analyzed by SDS-PAGE under reducing conditions. (A) Lysates were IP’d with anti-gH MAbs: 14-4b or AP86. (B) Lysates were IP’d with rabbit anti-peptide sera specific for gL in the absence of the gL peptide used to produce the antibodies (left panel) or with that peptide present in the IP (right panel). (C) Lysates were IP’d with the human anti-gB MAb 758. Molecular mass (MW) markers are indicated on the left.

We expressed HCMV glycoprotein gB from either the clinical strain TR or the lab strain AD169 along with TR gH/gL using non-replicating (E1-) adenovirus (Ad) vectors in ARPE-19 epithelial cells. The cells were labeled with ^35^S-methionine/cysteine, lysed in 1% Nonidet P-40 (NP-40), and the lysates were subjected to immunoprecipitation (IP) using gH-specific monoclonal antibodies (MAbs) and then analyzed by SDS-PAGE under reducing conditions. IP with the gH-specific MAbs 14-4b or AP-86 produced bands representing 85-kDa gH, 30-kDa gL, as well as a weaker 116-kDa band representing gB (Fig 1A). The amount of gB co-IP’d with MAb 14-4b was higher than MAb AP86 and perhaps is related to the fact that MAb 14-4b recognizes a conformational-dependent epitope, while MAb AP86 recognizes a linear epitope. Similar IPs were performed with a rabbit anti-gL peptide sera that precipitated the 85-kDa gH polypeptide, the 30-kDa gL polypeptide, as well as the 116-kDa gB polypeptide (Fig 1B). Addition of the gL peptide in the IP reaction reduced IP of gL and gH and also abolished the co-precipitation of gB (Fig 1B). Reciprocal IPs were also performed with radiolabeled extracts using the anti-human gB MAb 758. This gB MAb precipitated a band representing the 116-kDa gB along with a weaker band representing 85-kDa gH (Fig 1C).

**Fig 2:**
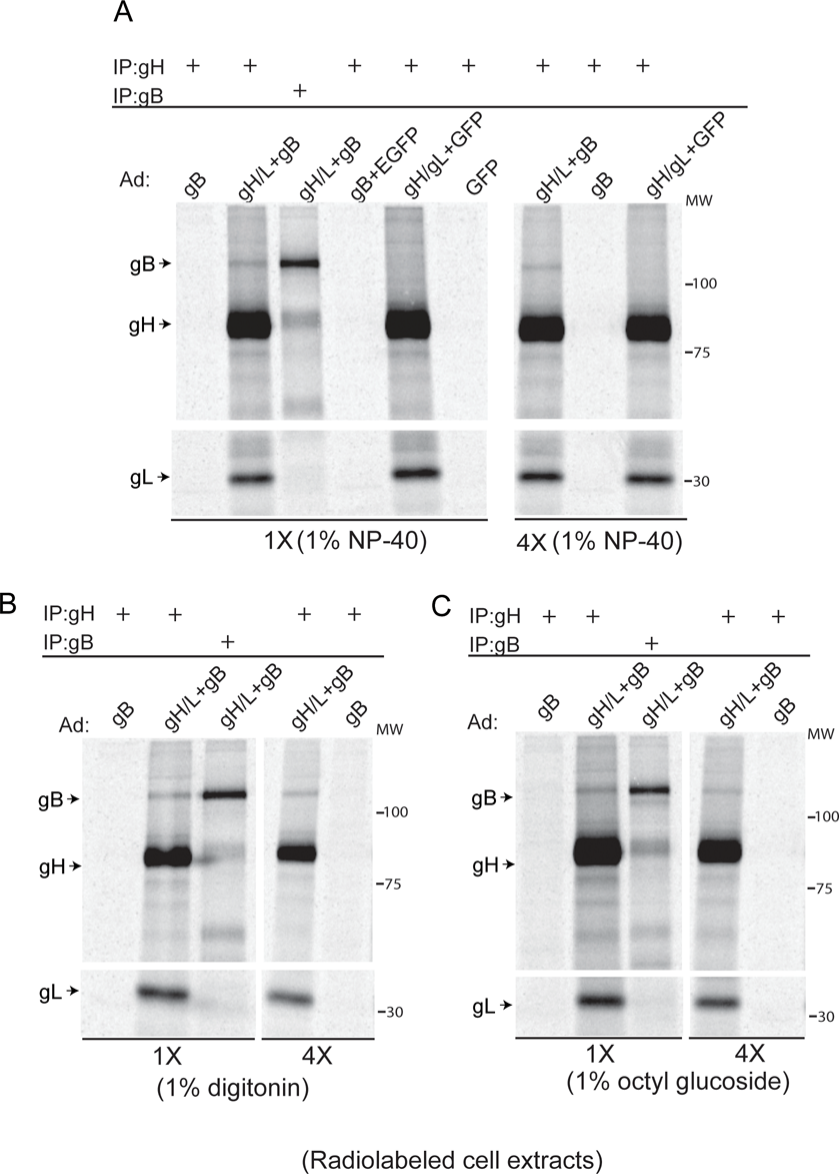
Different detergents and dilution of cell extracts does not destabilize gB-gH/gL complexes. (A) ARPE-19 cells were transduced with Ad vectors expressing gB, gB and gH/gL, or gH/gL and GFP for 20 hrs then radiolabeled for 4 hrs. Cell extract were made using IP buffer containing 1% NP-40. Some of each extract was then diluted with 4 times the volume of the same buffer (4X). Proteins were precipitated from cell extracts using either anti-gB MAb 27–156 or anti-gH MAb 14-4b then proteins analyzed by SDS-PAGE under reducing conditions. (B) ARPE-19 cells were transduced as described for panel A and extracts were prepared with IP buffer containing 1% digitonin. IP’s were performed with either anti-gB MAb 27–156 or anti-gH MAb 14-4b with undiluted samples (1X) or after samples were diluted with 4 times the volume (4X) of IP buffer containing 1% digitonin. (C) IPs were performed as described for panel B except cell lysates were prepared and diluted with IP buffer containing 1% octyl glucoside. The antibodies used for the IPs and the Ad vectors used to transduce cells are indicated above each panel. The detergents used in the IPs and whether samples were used neat or diluted is indicated below each panel. The positions of gB and gH/gL are indicated on the left side of the panels and molecular mass markers (MW) are indicated on the right.

We also performed IP experiments from radiolabeled ARPE-19 cells with buffers containing different detergents to rule out non-selective IP of proteins due to micelle formation or other aggregates. IPs performed using 1% digitonin or 1% octyl glucoside resulted in co-IP of gB with gH/gL similar to IPs performed with buffer containing 1% NP-40 (Fig 2A–2C). These experiments were repeated after 4-fold dilution of the cell extracts. The results of these experiments showed no major reduction in the amount of gB that was co-IP’d with gH/gL from the diluted samples (4X) when compared to the undiluted IP’s (1X), confirming that the association of gB with gH/gL is specific and sufficiently stable to withstand dilution (Fig 2A–2C). We noted that the amount of gB that was co-IP’d with gH/gL varied. In some cases, anti-gH MAbs co-IP’d as much gB as anti-gB MAbs (Fig 1A and 1C), whereas in other cases it was much less (Fig 2A–2C). We also noted that gB expression was higher from the AD169-gB Ad vector compared to the TR-gB Ad vector (Fig 1A and 1C) and attribute this to earlier versus later generation Ad construction (e.g. different transfer and Ad plasmids that could contain variations in c*is*-acting sequences within promoters). Thus, for all subsequent Ad transductions, we used the AD169-gB Ad vector.

### Characterization of gB-gH/gL complexes using IP/western blots

**Fig 3:**
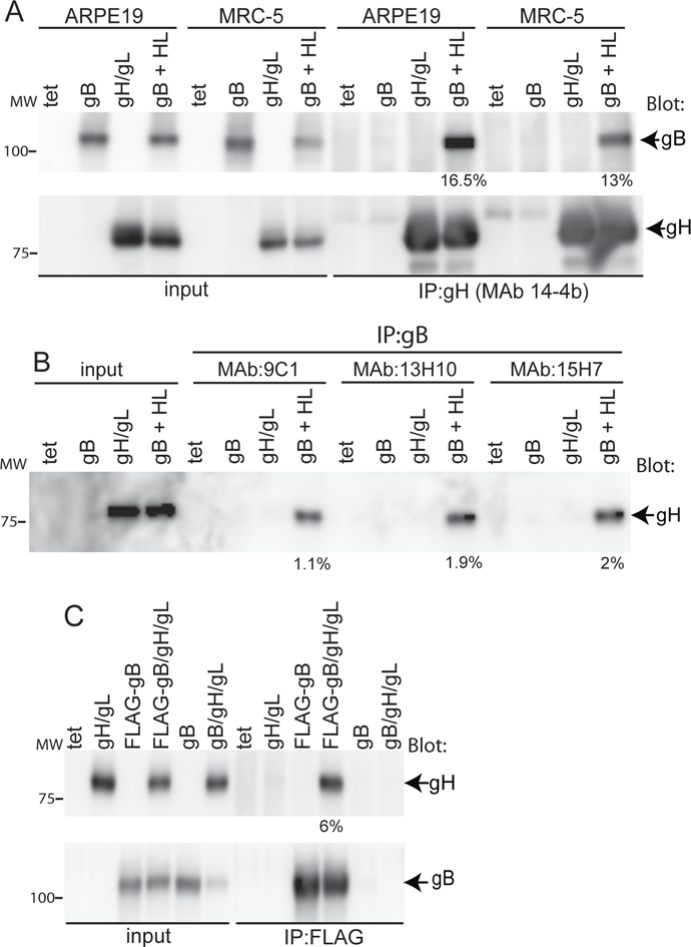
HCMV gB-gH/gL complexes detected by IP-western blots. ARPE-19 or MRC-5 cells were transduced with Ad vectors expressing HCMV gB, gH/gL, both gB and gH/gL, or with Ad-tet-trans (tet, as a negative control) as indicated at the top part of each panel. After 20 hrs the cells were lysed in IP buffer containing 1% NP-40. (A) Proteins were IP’d from ARPE-19 or MRC-5 cell extracts with anti-gH MAb 14-4b, separated by SDS-PAGE under reducing conditions, transferred to membranes, and then analyzed by western blot using rabbit polyclonal sera specific to gB or anti-gH MAb AP86. (B) Proteins from ARPE-19 cell lysates were IP’d with anti-gB MAbs 9C1, 13H10, or 15H7 and the IPs analyzed by western blot as described above using anti-gH AP86. Input represents 5% of the extract loaded directly onto gels then blotted. (C) ARPE-19 cells were transfected with an Ad vector expressing gB with a C-terminal FLAG epitope tag or wild type gB and co-transduced with Ad vectors to express gH/gL as indicated along the top of the panel. Proteins were IP’d with an anti-FLAG antibody and analyzed by western blot as described above with rabbit polyclonal gB-specific serum or anti-gH MAb AP-86 to detect gB and gH, respectively. The percent of gB and gH/gL that was co-IP in these experiments was quantified using NIH ImageJ software by comparing the relative band intensities from the IP’d proteins to the 5% input and are indicated under the lanes. Molecular mass (MW) markers are indicated on the left.

ARPE-19 epithelial cells or MRC-5 fibroblasts were transduced with different combinations of Ad vectors expressing gB and gH/gL and proteins IP’d from 1% NP-40 cell extracts with anti-gH MAb 14-4b. Proteins were transferred to membranes after SDS PAGE under reducing conditions and blotted with anti-gB rabbit serum or anti-gH MAb AP86. Under these conditions, we were able to detect gB in the gH/gL IP from extracts of APRE-19 and MRC-5 fibroblasts co-expressing gB and gH/gL (Fig 3A). Similar IPs performed with the gH-specific MAb with cells expressing only gB or only gH/gL did not co-precipitate gB (Fig 3A). Quantification of the amount of gB that was co-IP’d with gH/gL indicated that 16.5% and 13% of the total gB was precipitated with gH/gL from ARPE-19 or MRC-5 cells, respectively (Fig 3A).

Although we were able to IP gH/gL with gB using the human gB-specific MAb 738 from radiolabeled ARPE-19 cells (Fig 1C), several other gB antibodies including anti-gB MAb 27–156, did not co-IP gH/gL. One possibility is that certain gB antibodies such as 27–156 recognize a gB epitiope that is obscured by gH/gL binding. Thus, we produced a panel of new gB-specific MAbs by immunizing mice with a soluble form of gB [19] and screening for antibodies that were not blocked for binding to gB that was fixed on plates and pre-incubated with MAb 27–156. IP-western experiments showed that at least three of our new gB MAbs were able to co-IP gH/gL from Ad-transduced APRE-19 cells (Fig 3B). To further characterize the interaction, ARPE-19 cells were transduced with an Ad vector expressing gB with a C-terminal FLAG epitope tag (FLAG-gB). Western blot analysis of IP’s from cell lysates showed that an anti-FLAG MAb was able to co-IP gH/gL only from cells co-expressing FLAG-gB with gH/gL (Fig 3C). No gH/gL or untagged gB was detected from anti-FLAG IP’s even though the untagged Ad gB was transduced at the same MOI as Ad gB-FLAG. We noted that the ability to co-IP gH/gL with gB MAbs or anti-FLAG MAbs was still less efficient (1–6%) than co-IPs performed with gH MAbs.

**Fig 4:**
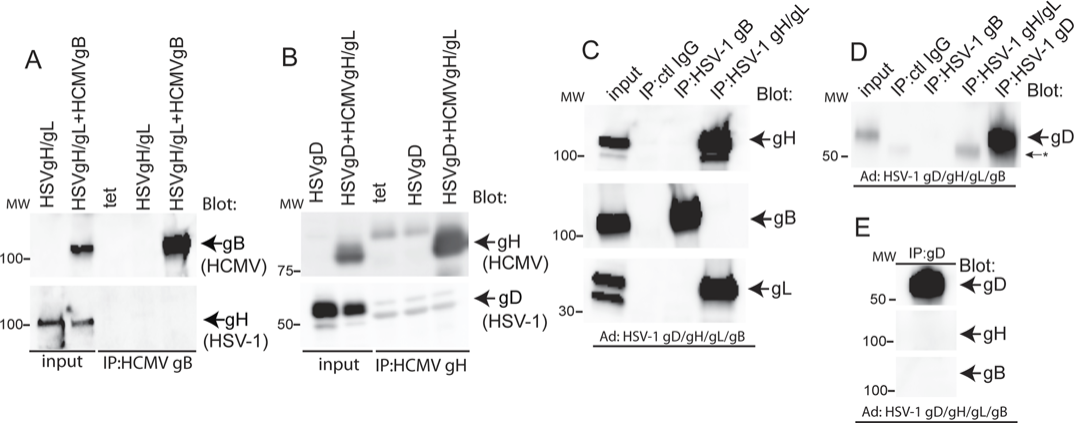
HSV-1 glycoproteins do not interact with HCMV glycoproteins. (A) ARPE-19 cells were transduced with Ad vectors expressing Ad-tet-trans alone (tet, as a negative control), HSV-1 gH/gL, or HSV-1 gH/gL along with HCMV gB (as indicated along the top of the panel). HCMV gB was IP’d from cell lysates with MAb 15H7 then blots probed with HCMV gB polyclonal serum (upper panel) or anti-HSV-1 gH polyclonal serum R137 (lower panel). (B) ARPE-19 cells were transduced with Ad vectors expressing HSV-1 gD alone or with HSV-1 gD and HCMV gH/gL or with Ad-tet-trans (tet, a negative control) then lysates IP’d with HCMV gH/gL-specific MAb 14-4b. Proteins were analyzed by SDS-PAGE under reducing conditions and western blots were probed with rabbit anti-HSV-1 gD polyclonal serum (R45) or anti-HCMV gH MAb AP86. (C) ARPE-19 cells were transduced with Ad vectors expressing HSV-1 gB, gH/gL, gD, and nectin-1. After 24 hrs proteins were IP’d from cell lysates with anti-HSV-1 gB MAb SS10, anti-HSV-1 gH MAb 53S, or irrelevant control MAb (15H7). The IP’d proteins were separated by SDS-PAGE under reducing conditions, transferred to membranes, and blotted with polyclonal rabbit serum R137 or R67 against HSV-1 gH/gL and gB, respectively. (D) Proteins were IP’d from ARPE-19 lysates with anti-HSV-1 gB MAb SS10, anti-HSV-1 gH MAb 53S, anti-HSV-1 gD MAb DL6, or irrelevant control MAb (15H7). The proteins were separated by SDS-PAGE under reducing conditions, transferred to membranes and blotted with rabbit anti-HSV-1 gD polyclonal serum (R45). The arrow with asterisk indicates cross reactivity of R45 with IgG heavy chain. (E) Proteins were IP’d from cell lysates, separated by SDS-PAGE and transferred to membranes as described above and then probed with rabbit polyclonal serum R45, R137, or R67 against HSV-1 gD, gH/gL, and gB, respectively. Input represents 5% of the total amount of lysate used for the IPs. Molecular mass (MW) markers are indicated on the left.

To further characterize the specificity of our IP-western experiments, we performed IPs with extracts from cells co-expressing HCMV and HSV-1 glycoproteins. When HSV-1 gH/gL was co-expressed with HCMV gB and HCMV gB IP’d with the anti-HCMV gB MAb 15H7, no HSV-1 gH was co-IP’d (Fig 4A). Similarly, co-expression of HSV-1 gD with HCMV gH/gL followed by IP of HCMV gH/gL using MAb 14-4b did not show evidence for co-precipitation of HSV-1 gD by western blot analyses (Fig 4B). We also attempted to provide evidence for complexes of HSV-1 glycoproteins in Ad-transduced cells. ARPE-19 cells were transduced with Ad vectors expressing HSV-1 gD, gH, gL, and gB along with an Ad vector expressing the cellular receptor nectin-1. At 24 hrs after transduction, most of the cells in the monolayer were undergoing fusion, similar to what we observe with HCMV glycoproteins. At this point, 1% NP-40 extracts of cells were made and HSV-1 gH/gL and gB proteins IP’d and analyzed by western blot. IP of HSV-1 gH/gL with MAb 53S showed no evidence of gB and IP of HSV-1 gB with MAb SS10 showed no evidence of HSV-1 gH/gL (Fig 4C). We also tested whether IP of HSV-1 gB and gH/gL co-IP’d HSV-1 gD. Western blot analyses of these IPs provided no evidence that HSV-1 gD could co-IP with HSV-1 gB or gH/gL (Fig 4D). Moreover, reciprocal IP’s performed with the anti-gD MAb DL6 also failed to provide evidence that HSV-1 gB or gH/gL could be co-IP’d with HSV-1 gD (Fig 4E).

### gB and gH/gL form complexes form early after synthesis

**Fig 5:**
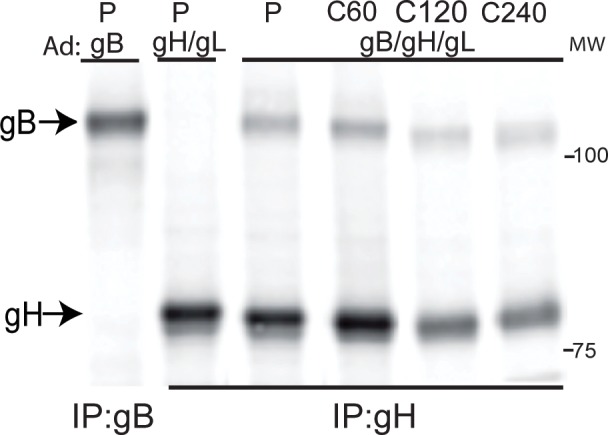
gB-gH/gL complexes form early after synthesis and are stable. ARPE-19 cells were transduced with Ad vectors expressing either gB or gH/gL alone or with a combination of both gB and gH/gL. After 24 hrs, the cells were radiolabeled for 15 min. with ^35^S-methionine/cysteine then 1% NP-40 cell extracts made immediately (P) or the radiolabeled cells incubated in chase media with excess non-radioactive methionine and cysteine, i.e. the label chased (C) for 60, 120, or 240 min. After these chase periods, cell extracts were made immediately and proteins IP’d with anti-gH MAb 14-4b (IP: gH) or anti-gB rabbit serum (IP:gB) and analyzed by SDS-PAGE under reducing conditions. Molecular mass (MW) markers are indicated on the right.

Pulse-chase experiments were performed to characterize the kinetics of gB-gH/gL complex formation using Ad-transduced cells. ARPE-19 cells were transduced with Ad vectors expressing gB and gH/gL for 20–24 hr then the cells were radiolabeled with ^35^S-methionine/cysteine for 15 min and the label chased for 60, 120 or 240 min. and the proteins analyzed by SDS-PAGE under reducing conditions. In the 15 min pulse sample, gB was co-IP’d with gH/gL and this quantity of gB remained stable for 60 min before slowly declining with about half remaining even after 240 min (Fig 5). Therefore, gB-gH/gL complexes form after synthesis in the ER and are relatively stable. Note that the quantity of gB complexed with gH/gL was ~38% of the gH/gL precipitated in the pulse sample from the transduced cells.

### gB and gH/gL complexes form in HCMV-infected cells and are found in virions

**Fig 6:**
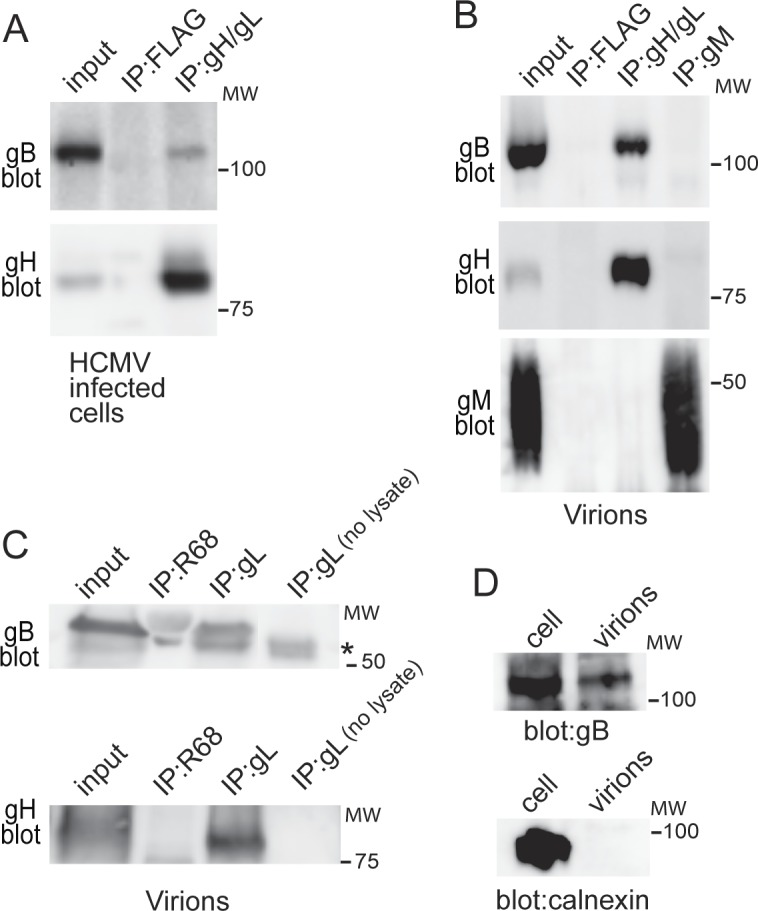
gB-gH/gL complexes in HCMV-infected cells and extracellular virions. (A) Human fibroblasts were infected with HCMV BADrUL131 for 3 days then cells were pelleted and cell extracts made using 1% NP-40. gH/gL was IP’d using MAb 14-4b or cell extracts incubated with an irrelevant MAb specific for FLAG (no FLAG epitope was present in gH/gL). The IP’d proteins were then subjected to SDS-PAGE under reducing conditions and analyzed by western blot using rabbit gB-specific antiserum (gB blot) or anti-gH MAb AP86 (gH blot). (B) HCMV extracellular particles partially purified from 10-day HCMV infected fibroblast culture supernatants were solubilized in 1% NP-40 and the proteins IP’d with MAb anti-FLAG (irrelevant MAb), anti-gH 14-4b, or an anti-gM MAb. The IP’d proteins were analyzed by western blot as described above using rabbit anti-gB polyclonal sera, anti-gH MAb AP86, or a gM MAb. (C) Proteins from solubilized extracellular virus particles were IP’d with rabbit anti-gL peptide serum (IP:gL), a control antibody to HSV-1 gB (IP:R68) or rabbit anti-gL peptide serum alone with no HCMV lysate (IP:gL no lysate) to control for cross reactivity of the IgG in the serum with anti-rabbit secondary antibodies. The proteins were analyzed by western blot and membranes were probed with anti-HCMV gB MAb 27–156 (gB blot, upper panel) or with rabbit anti-gH peptide serum (gH blot, lower panel). Migration of the rabbit IgG which was picked up in the in the gB blot is indicated by the asterisk. In panels A, B, and C, input represents 5% of the total amount of lysate. (D) An equal amount of cell-associated virus stock (cell) or virus stock purified from cell supernatants (virions) was separated by SDS-PAGE, transferred to membranes and blotted with anti-HCMV gB MAb 15H7 or a polyclonal rabbit antibody to calnexin. Molecular mass (MW) markers are indicated on the right.

The results to this point involved expression of just gB and gH/gL using non-replicating Ad vectors. To assess complexes in HCMV-infected cells, human fibroblasts were infected with HCMV BAD*r*UL131 [20] and at 3 days post-infection cell extracts were prepared with 1% NP-40. gH/gL was IP’d using MAb 14-4b or an irrelevant MAb specific for FLAG (used as a negative control) then subjected to western blotting with gB or gH antibodies. The results of these IPs showed that gH antibodies could co-IP gB with gH/gL from HCMV infected cells (Fig 6A). To detect gB-gH/gL complexes in virions, extracellular HCMV virions were purified from cell culture supernatants of fibroblasts infected with HCMV BAD*r*UL131 at 10 days post-infection. The virus particles were solubilized in 1% NP-40 and proteins IP’d with gH-specific MAb 14-4b, an irrelevant anti-FLAG antibody, or a MAb specific for the HCMV glycoprotein M (gM) that is present in the virion. Western blots analysis of the IPs under reducing conditions showed that gB was co-IP’d along with gH/gL, amounting to 2.6% of the total gB in the detergent extracts (Fig 6B). It is not yet clear that MAb 14-4b precipitates all of the gB present in the gB-gH/gL complex because a much higher fraction of gH/gL is present in these complexes (see next section). It is also important to note that anti-gH MAb 14-4b also recognizes trimer (gH/gL/gO) and pentamer (gH/gL/UL128-131) complexes, which might compete with binding to gB-gH/gL complexes (see below). Lysates from extracellular HCMV particles were also subjected to IP using rabbit anti-gL peptide serum. Similar to IP’s with antibodies to gH, the rabbit anti-gL serum also co-precipitated gB and this amounted to about 2.5% (Fig 6C). However we also blotted the gL IP for gH and noted that only about 8% of the total gH was IP’d with gL antibodies, suggesting that our anti-gL peptide serum does not efficiently IP gH/gL complexes from virion lysates (Fig 6C). Confirmation that our virus preparations were not contaminated with cellular proteins was confirmed by blotting for the cellular protein calnexin, which did not show calnexin in virion preparations (Fig 6D). We concluded that gB-gH/gL complexes are found in HCMV infected cells and in extracellular virus particles.

## gB interacts with gH/gL but not gH/gL/gO or gH/gL/UL128-131

**Fig 7:**
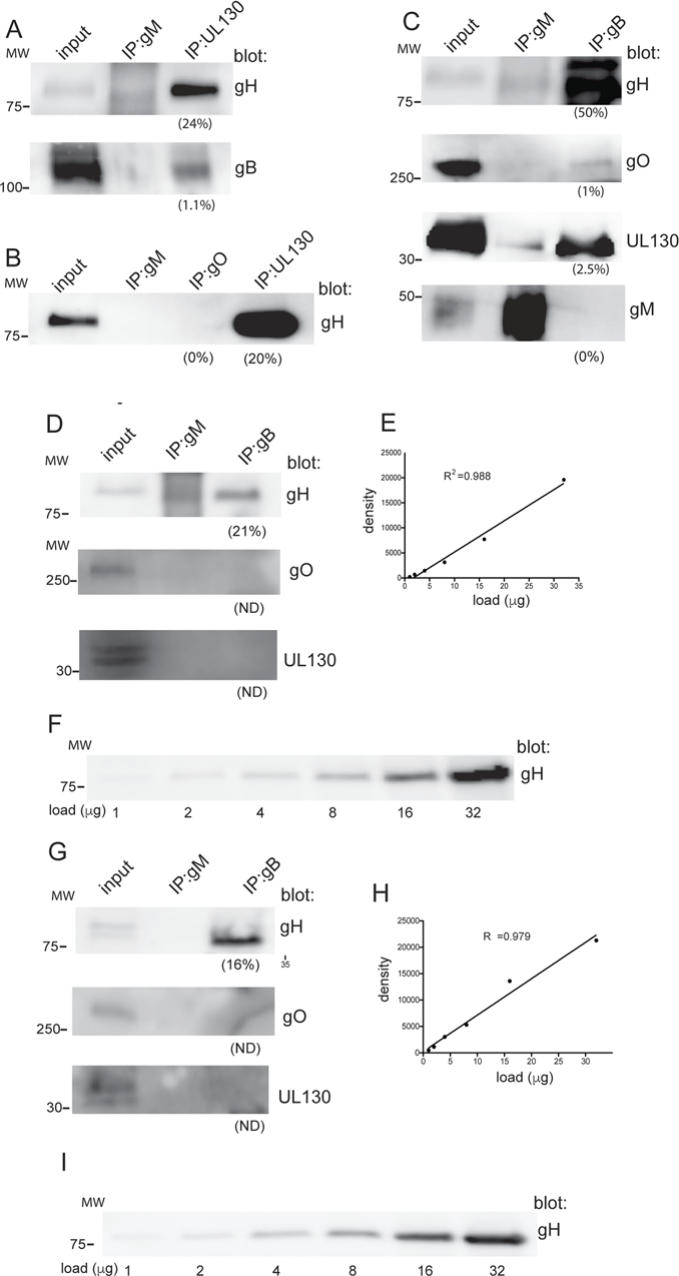
HCMV gH/gL/gO and gH/gL/UL128-131 do not significantly complex with gB in virions. HCMV BADrUL131 extracellular virus particles were partially purified from cell supernatants harvested10 days post -infection then solubilized in 1% NP-40 and insoluble proteins removed by centrifugation. (A) Proteins from virion extracts were IP’d using rabbit polyclonal anti-peptide antibodies specific for UL130 or a gM-specific MAb as a negative control and the IP’d proteins separated by SDS-PAGE and analyzed by western blot with gH-specific MAb AP86 or gB-specific MAb 15H7. (B) Proteins were IP’d from detergent extracts of HCMV virions with rabbit polyclonal antibodies specific for UL130, rabbit polyclonal anti-peptide serum specific for gO (TBgO) [23] or with a gM-specific MAb and the IP’s analyzed by western blot as described above with the gH-specific MAb AP86. (C) Detergent extracts of extracellular HCMV virus particles were solubilized then extracts IP’d with anti-gM MAb or anti-gB MAb 15H7 (as indicated along the top of the panel). The precipitated proteins were analyzed by western blot with anti-gH MAb AP86, rabbit anti-gO (TBgO) sera, rabbit anti-UL130 sera, or a gM-specific MAb (as indicated along the right side of the panel). (D) IP-western blot analyses of solubilized BADrUL131 extracellular virions as described for panel C, except that the membrane blotted for gH from the gB IP was exposed along side a linear range of input samples derived from a gH/gL expressing cell lysate. (E) Linear curve of the gH signal generated after exposure of the membrane containing the increasing doses of the gH-gL expressing lysate. The y-axis indicates the relative density of the protein bands and the x-axis indicates the amount of lysate loaded into each well. (F) Shown is the exposure of the western blot containing the increasing amounts of the gH-gL expressing lysate. The amount to gH/gL expressing lysate loaded into each well is indicated below the panel. (G) Western blot analyses as described for panel D except IP’s were performed with solubilized extracellular virions derived from the clinical strain TR. (H) Linear curve of the gH signal containing the increasing doses of the gH-gL expressing lysate as described for panel E. (I) Shown is the exposure of the western blot containing the increasing amounts of the gH-gL expressing lysate. The amount to gH/gL expressing lysate loaded into each well is indicated below the panel. To assess the relative quantity of IP’d proteins, we compared the signal intensities of the IP’d proteins to the input protein signal intensities. Analysis was performed using ImageJ software (panel C) or Image Studio software (Licor) for panels 7D-7I. For all blots, input refers to 5% of the virion lysate loaded directly into gels. The percent of the total protein IP’d compared with the total in the virion extract is shown under each lane. Molecular mass (MW) markers are indicated on the left. All samples were analyzed by SDS-PAGE under reducing conditions with the exception of samples involving the detection of gO, which required that that SDS-PAGE be performed under non-reducing conditions, thus the signal for gO represents the gH/gL/gO disulfide linked >250 kDa trimer. ND indicates not detected.

HCMV virions contain at least two forms of gH/gL: a trimer of gH/gL/gO and a pentamer of gH/gL/UL128-131 [5]. Thus it was important to establish whether these forms of gH/gL are complexed with gB in virus particles. Initially, we IP’d proteins from detergent extracts of extracellular HCMV BAD*r*UL131 virions with a rabbit anti-UL130 polyclonal serum that IPs the gH/gL/UL128-131 pentamer [21]. When these IPs were analyzed by western blot using anti-gB rabbit serum, only a small fraction (~1%) of the total gB present in the virions was precipitated (Fig 7A). In contrast, the UL130 antibody precipitated ~24% of the total gH/gL. Previously, we and others have experienced difficulties using existing gO-specific anti-peptide antibodies to detect gO from virions [22,23]. These gO antibodies function well in western blots but only when samples are subjected to electrophoresis under non-reducing conditions. In the present experiments, IP of HCMV virions with a gO specific polyclonal anti-peptide antibody failed to precipitate any detectable quantity of gH/gL when analyzed by western blot, (Fig 7B), again suggesting that these gO anti-peptide sera do not recognize gO in virions. Unfortunately, because additional antibodies to gO are not yet available, we were unable to IP gH/gL/gO-specific complexes to assess co-IP of gB as in Fig 7A.

As an alternative approach, we IP’d proteins from detergent extracts of virions with the high affinity gB MAb 15H7 then western blotted for gH/gL, UL130, or gO. A control MAb specific for glycoprotein gM was also included. The IP’d proteins were separated by SDS-PAGE under reducing conditions except for IP’s involving western blots to detect gO, which involved non-reducing conditions. When western blots of proteins IP’d with the gB-specific MAb were probed with the anti-gH MAb AP86, the results clearly showed co-IP of gH/gL. We then evaluated the signal intensity between the IP’d gH/gL and the 5% input and this indicated that ~50% of the total gH/gL was complexed with gB (Fig 7C). Similar analyses were performed with blots probed with gO and UL130 antibodies and in contrast, very little gO (1%) or UL130 (2.5%) was co-IP’d with gB (Fig 7C). We also did not detect gM in the gB IP as expected (Fig 7C). These IP-western blot experiments were repeated with additional virus preps generated from BADrUL131 (Fig 7D) and from the HCMV clinical strain, TR, (Fig 7G). In addition, we analyzed blots containing increasing concentrations of gH/gL cell lysates allowing us to define the linear dynamic range of our western blot signals and ensure our signals were not over-exposed to the point of saturation. Western blot analyses of the gH that was co-IP’d from BADrUL131 virions under these conditions showed that 21% of the total gH was co-IP’d with gB (Fig 7D). Blots of the gH that co-IP’d with gB from TR derived virions showed 16% of the total gH co-IP’d with gB (Fig 7G). In both cases, the gH blot signals fell within the linear range of detection established by simultaneous exposure of the gH/gL cell extracts (Fig 7D–7F and 7G–7I). In these particular experiments involving BADrUL131 and TR virions, we were unable to detect any gO or UL130 that was co-IP’d with gB (Fig 7D and 7G). The range of gH that was co-IP’d with gB in these experiments (16%-50%) was well within the expected range, given the heterogeneity of virus particles for glycoproteins and strain differences. We concluded that gH/gL/gO and gH/gL/UL128-131complexes are not associated with significant fractions of gB in HCMV virus particles, whereas a large fraction of gH/gL lacking gO or UL128-131 is complexed with gB.

### HCMV gB fusion loop mutants and gH/gL mutants interact to form gB-gH/gL complexes

**Fig 8:**
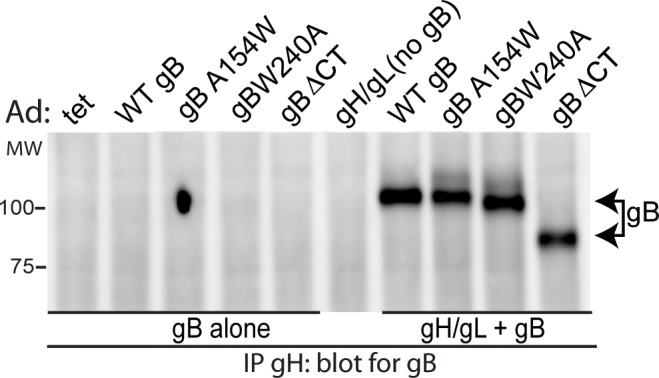
Fusion loop gB mutants complex with gH/gL. ARPE-19 cells were transduced with Ad vectors expressing wild type gB (WT gB) or mutant forms of gB: gBA154W, gBW240A, or gBΔCT alone or in combination with gH/gL. Extracts were made using 1% NP-40 and gH/gL IP’d from extracts using MAb 14-4b. Western blots were probed with anti-gB rabbit polyclonal serum.

We found HCMV gB-gH/gL complexes in extracellular virions suggesting that these complexes form before gB is activated for fusion and entry. What was of interest here was whether HCMV gB must be fusion competent in order to interact with gH/gL. With HSV-1, gB “fusion loop” mutants did not interact with gH/gL in BiMC assays and BiMC fluorescence was only observed around sites of cell-cell fusion [6]. We previously described the HCMV gB mutants gBA154W and gBW240A [24], that contain single substitutions in gB domains which are predicted to be homologous to EBV and HSV-1 fusion loops [25]. Using cell-cell fusion assays, gBA154W and gBW240A did not promote cell-cell fusion and were unable to promote entry in trans [24]. To determine whether these gB mutants could interact with gH/gL, ARPE-19 epithelial cells were transduced with Ad vectors to express wild type gB, gBA154W, or gBW240A along with wild-type gH/gL and then proteins were IP’d from the cell extracts using MAb 14-4b. Western blots probed with an anti-gB-specific rabbit serum showed that the gB mutants gBA154W and gBW240A formed complexes with gH/gL (Fig 8A). A third HCMV gB construct, gBΔCT, that lacks the entire cytoplasmic domain of gB and displays no cell-cell fusion activity [18,24] also interacted with gH/gL (Fig 8A). These observations fit well with the results above that indicate gB-gH/gL complexes form early after synthesis and are incorporated into the virion and clearly indicate that gB-gH/gL complexes form even when gB is not capable of fusion.

To attempt to identify gH/gL residues that make contact with gB, we made several mutations in gH and gL involving clusters of linear 2–5 amino acid sequences of HCMV gH and gL that were predicted to be on protein surfaces based on the homology with the EBV and HSV-1 gH/gL structures. Our rough predictions of the structures of HCMV TR gH/gL involved comparisons to HSV-1 and EBV gH/gL sequences using the RaptorX Structure alignment server (http://raptorx.uchicago.edu/DeepAlign). This produced a crude 3D structure of HCMV gH/gL that was used along with primary sequence alignments between HCMV, HSV-1, and EBV gH/gL molecules [25] to design mutations in HCMV gH/gL that were likely to affect protein surfaces and yet avoid disrupting gH/gL dimer formation or produce gross misfolding. We made fewer mutations near the N-terminal of gH given that this region is where EBV and HSV-1 gH binds gL [25,26]. A total of 42 mutations in the gH polypeptide and 20 mutations in gL polypeptide were made, in each case, replacing the existing residues with alanine (S1 and S2 Tables). All the gH mutants including the wild-type gH control were fused to a C-terminal FLAG epitope tag to ensure that we could IP gH (without using the conformation-specific gH MAb 14-4b). Mutant gH and wild type gH (with FLAG) proteins were co-expressed with wild-type or mutant gL in conjunction with gB by plasmid transfection into 293T cells. The cells were radiolabeled at 48 hr post-transfection and proteins IP’d from detergent extracts using a FLAG-specific MAb. For all the mutants, heterodimeric gH/gL complexes were detected (S1 and S2 Tables). In fact, none of the mutants we predicted using the methods above produced unstable gH or gL molecules. Moreover, all of the gH and gL mutants assembled complexes with gB (S1 and S2 Tables). We note that one of these mutants involved the gL cysteine-144 that has been shown to abolish interactions with gO and UL128 [27], suggesting that unlike trimer and pentamer complexes, this residue is not involved in gB-gH/gL complex formation (S2 Table). One might characterize these results as negative data, but we would suggest that these mutants will shape further studies of gB-gH/gL interactions. The results also suggest that the surfaces of gH/gL that interact with gB include discontinuous sequences (more than a single continuous polypeptide chain), so that gB-gH/gL interactions are not abolished by simple mutations involving small substitutions. These results also fit with the stability of the gB-gH/gL complexes. These data also indicate that the interaction of gB with gH/gL does not entail the same disulfide bonds formed between gO, UL128-131, and gH/gL complexes.

## Discussion

All herpesviruses express gH/gL and gB homologues and for most, it is clear that both glycoproteins are absolutely required for entry. It has been frequently suggested that herpesvirus gH/gL molecules interact with gB to trigger gB fusion activity and virus entry. Support for these models largely involves studies with HSV-1 involving bimolecular complementation in transfected cells and *in vitro* assays of soluble glycoproteins binding to liposomes [7,8,10]. These methods sought to explain the need for both HSV-1 gH/gL and gB in entry, but none of the data produced strong support for direct interactions of gH/gL with gB in virus-infected cells or virions. There has been no evidence of any interactions between gB and gH/gL in γ- or β-herpesviruses.

Here, we describe a stable complex formed between HCMV gB and gH/gL that we observed under a variety of expression conditions, using different detergents, following dilution of extracts, and using a panel of gH-, gL-, gB-, and epitope tag-specific antibodies. As further evidence of specificity, HSV-1 gH/gL and gD not interact with HCMV gB or gH/gL and HCMV gH/gL did not co-IP with gM from virions. HCMV gB-gH/gL complexes were produced early after protein synthesis in the ER in Ad-transduced cells and assembled without other HCMV proteins. Moreover, gB fusion loop mutants and truncated forms of gB that lack fusion activity interacted well with gH/gL. These observations fit together well if one considers the gB-gH/gL complex forming early, being incorporated into virions and then ultimately acting in membrane fusion (during virus entry) after virus is release from cells and binds onto cell surfaces.

Importantly, gB-gH/gL complexes were detected in HCMV infected cells and in extracellular virions, amounting to 16–50% of the total gH/gL in the virion (Fig 7C, 7D and 7H). The quantities of gB that was IP’d with gH/gL from detergent extracts of virions were lower, 2.5–2.6% in the experiments shown (Fig 6B and 6C). However, it is important to note that various conditions involving IP’s of gH/gL from Ad-transduced cells produced a larger fraction of the gB present in gB-gH/gL complexes. In cells radiolabeled following Ad-transduction, the signal for gB was ~38% of gH/gL signal (Fig 5). In experiments in which gH/gL was IP’d from Ad transduced cells and blotted with gB Abs, 13–16% of gB co-IP’d with gH/gL (Fig 3A) and 6% of gH/gL co-IP’d with gB (Fig 3C). There are at least three explanations for these differences between the quantities of gB complexed with gH/gL in virions versus those involving Ad-transduced cells. First, in virions there are three forms of gH/gL complexes, gH/gL, gH/gL/gO, and gH/gL/UL128-131, and the gH/gL antibodies used here for IPs recognize all three species. gH/gL/gO and gH/gL/UL128-131 were not expressed in Ad-transduced cells. Thus, there may be competition for binding of different gH/gL forms that reduce the quantities of gB-gH/gL complexes detected from virions. Second, we have a limited number of gH/gL MAbs and it could well be that those we used inefficiently recognize the form of gH/gL that is complexed with gB. In Ad-transduced cells (where more gB is IP’d) the cells are undergoing gB-gH/gL-mediated cell-cell fusion. Thus, at any given time in Ad-transduced cells, there may be pre-fusion, fusion, and post-fusion forms of these glycoproteins and the ratios of these different forms is likely very dynamic over time and might vary substantially in different experiments. gH/gL is just as likely to be undergoing conformational changes as is the fusion protein gB. By contrast, most of the gH/gL and gB glycoproteins found in virions must be in the pre-fusion form if viruses are to be able to enter cells. These conformational changes are likely to have a significant effect on the capacity of antibodies to IP gB, gH/gL and the gB-gH/gL complexes. Thus, our gH/gL antibodies may prefer the conformational changes in gH/gL that are associated with membrane fusion so that we obtain more gB-gH/gL complexes from Ad-transduced cells compared with virions. Third, it is important to note that IPs are rarely efficient. For example, it is often the case that an equal amount of antigen can be IP’d from the supernatants after the initial IP is performed (e.g. IP then re-IP) suggesting that only a fraction of the total antigen can be immuno-captured. Related to this point, it is clear that different antibodies pull down quite different amounts of the gB-gH/gL complexes. (See for example, Fig 1A and 1B involving different gH and gL antibodies.). Different methods of expression and labeling conditions also likely produced different quantities of complexes. Nevertheless, our observations that 16–50% of the total gH/gL was complexed with gB in virions and as much as 38% of gB was complexed with gH/gL in Ad-transduced cells demonstrate that these complexes can form in a relatively efficient manner. Given that extracellular virions are only one snapshot of the virus life cycle and these pictures were taken with only a few antibodies, the maximum percentage of gB in these complexes might have been under estimated.

These observations are fundamentally important because this is the first solid evidence for direct gB-gH/gL interactions and, importantly, in virus-infected cells and virions. The previous support for HSV-1 gB-gH/gL interactions involving bimolecular complementation (BiMC) showed fluorescence and fusion. Related to points made in the last paragraph, there were no measurements of what fraction of gB interacted with gH/gL in these BiMC experiments. HCMV gB and gH/gL are sufficient for cell-cell fusion and are, thus, the minimal fusion machinery [13,18]. The presence of a gB-gH/gL complex in the HCMV virion suggests that this trimeric or multimeric structure might be involved in entry fusion, though we acknowledge that we have not yet established the functional significance of the gB-gH/gL complexes here. Nevertheless, our observations are highly significance because of the dogma, the prevailing premise that both gB and gH/gL are essential for entry and must therefore interact. As we said, the BiMC studies supported these interactions but did not shown them directly. Our observations that as much as 16–50% of virion gH/gL is complexed with gB, is strong support for the hypothesis that gB-gH/gL complexes act during virus entry.

Direct proof that gB-gH/gL complexes are essential for or play important roles in virus entry fusion or cell-cell fusion (i.e., functional significance) requires mutations that abolish complex formation. We constructed 62 mutants involving substitutions of 3–5 amino acids in gH and gL which were designed to alter putative surface residues but none abolished interaction of gH/gL with gB. The C-terminal and fusion loop mutants of gB also bound gH/gL. In order to definitively establish the functional significance of gB-gH/gL complexes, more mutants that abolish complexes must be made. This will likely require larger mutations in gH/gL or gB, e.g. affecting more than a single continuous sequence of gH or gL to prevent gB binding. The observation that gB-gH/gL complexes are relatively stable in detergents and in pulse-chase experiments suggests that extensive surfaces of these proteins might contact one another or that there are covalent interactions. Mutations to abolish these contacts require construction of larger mutations and are best done with the structures of the protein in hand. HCMV has lagged behind HSV and EBV in terms of glycoprotein structures, mapped MAb epitopes, and other reagents, so that the structure-function studies required to demonstrate functional significance extend far beyond the scope of this report.

A major advance derived from our observations differs from the current view of how HSV-1 gB and gH/gL proteins interact. The BiMC results suggested that HSV-1 gB and gH/gL are positioned as separate molecules in the virion envelope, but bind to one another only just before or during membrane fusion [8]. Blocking the fusion activity of gB (fusion loop mutations) abolished HSV-1 gB-gH/gL interactions. It appeared that HSV-1 gH/gL is altered by gD binding to receptors then transiently interacts with gB, but the nature of this and whether it indeed occurs is not clear. Consistent with this, we did not detect HSV-1 gB-gH/gL complexes in detergent extracts of Ad-transduced cells. By contrast, HCMV glycoproteins form stable HCMV gB-gH/gL complexes irrespective of gB fusion activity. This is considered fundamentally different from HSV-1. This difference may relate to the fact that HSV-1 expresses a separate receptor binding protein, gD, whereas gH/gL homologues in HCMV and EBV apparently bind receptors [4,5,28]. That said the differences between HSV-1 and HCMV fusion machineries might not be so great. Our studies could be viewed as suggesting a fusion model with a different starting point, i.e., HCMV gH/gL in pre-established complexes with gB in the virion triggers gB for fusion, whereas HSV-1 glycoproteins need to come together.

We were surprised that the gH/gL/gO trimer and gH/gL/UL128-131 pentamer complexes did not efficiently interact with gB in virions because these two forms of gH/gL have important functions in the virion envelope. Viruses that lack gO fail to enter all cells tested and UL128-131 are necessary for entry into many cell types (reviewed [5]). In one experiment, a gB MAb precipitated 50% of the gH from virions, but only 1–2% of the gH/gL/gO and gH/gL/UL128-131 complexes. In two other experiments, 16% and 21% of the gH was precipitated with a gB MAb and no detectable gH/gL/gO or gH/gL/UL128-131 was observed. Note that these results can not be compared to our observations that only 2.5% of the total gB in virions was in the complex, as the analyses for gH/gL/gO and gH/gL/ul128-131 was internally controlled, one gB MAb was used to IP, then blots were performed with gO, UL130, or gH MAbs, comparing the IP’d proteins to the total in the extract. Together, our IP assays argue for a gH/gL species, that is devoid of gO and UL128-131 that is in complex with gB. This is consistent with a previous report showing gH/gL heterodimers in the virion that are not disulfide liked to gO or UL128 [29]. In addition, a dimer of gH/gL heterodimers has been described in transfected cells but this gH/gL species has not yet been shown in virions [27]. Whether it is the gH/gL heterodimer or a dimer of gH/gL heterodimers that is bound to gB is not clear at this point and we have not ruled out other viral proteins present in the complex. Whether the gH/gL/gO trimer and gH/gL/UL128-131 pentamer interact with or “talk to” gB-gH/gL complexes during virus entry is not yet clear. Our previous interference studies suggested that gH/gL/gO binds receptors in fibroblasts, whereas gH/gL/UL128-131 binds epithelial and endothelial cell receptors [16,17]. gH/gL interfered poorly or not at all in these experiments. Recently, soluble gH/gL/UL128-131, but not soluble gH/gL, blocked HCMV entry into epithelial cells [30]. Thus, it seems possible or even likely that the trimer and pentamer complexes bind receptors and act upstream of entry fusion. It is not yet clear how this fits with observations that gH/gL and gB are sufficient for cell-cell fusion [13,18]. In cell-cell fusion, cells might be in close proximity without the requirements for certain types of receptor binding, and gH/gL and gB represent the core fusion machinery. Given that HCMV enters some cells at the plasma membrane and others after endocytosis, it is reasonable to believe that activation of the HCMV fusion machinery must be tightly regulated during entry but perhaps not during cell-cell fusion.

Observations that HCMV gH/gL interacts with gB in the ER may have implications for how gH/gL/gO and gH/gL/UL128-131 assemble. There was previous evidence for competition between gO and UL128-131 for binding to gH/gL [23]. This competition was recently explained by observations that a cysteine (C144) present in gL forms disulfide bonds with either gO or UL128 [27]. Our data raises the possibility that gB might also compete with UL128-131 and gO for binding to gH/gL. However, our mutagenesis of C144 in gL did not abolish binding of gH/gL with gB. Efforts to determine whether gB is covalently bound to gH/gL have been inconclusive to date because of the complex nature of high molecular weight forms of gB and gH/gL in virions: dimers, trimers, pentamers and blots of non-reduced samples were complex.

One intriguing model derived from our results suggests the notion that the gB found in complex with gH/gL might be in the pre-fusion form. This is supported by observations that virions contain gB-gH/gL complexes and since these glycoproteins are designed for virus entry, virions represent the biologically relevant population of these glycoproteins. Given that some or all of the gB in virions must be in the pre-fusion form in order for virus entry to occur, it is possible that gH/gL that is complexed with pre-fusion gB can respond to receptors or other stimuli then this gH/gL can trigger adjacent gB for fusion. However, we acknowledge that we have no direct evidence as to whether the gB associated with gH/gL in Ad-transduced cells or the virion is in the pre-fusion form. The structures of HSV-1 and EBV gB were derived from soluble proteins secreted from insect cells without gH/gL and were both apparently in the post-fusion forms [1,2]. In the virion or as soluble proteins, gH/gL might stabilize gB in the pre-fusion form. Thus, it might be possible to derive the structures of gB-gH/gL complexes in order to obtain the structure of the pre-fusion form of gB. This would have important implications for the development of HCMV vaccines and antibodies that block the transition from pre-fusion to post-fusion gB would likely be important. Previous efforts to develop subunit vaccines involved soluble forms of gB and were not successful [31].

## Materials and Methods

### Cells

Human MRC-5 embryonic lung fibroblasts and human retinal pigmented epithelial cells (ARPE-19) were obtained from American Type Culture Collection and cultured in Dulbecco’s modified Eagle’s medium (DMEM) supplemented with 10% fetal bovine serum (FBS) or DMEM/Ham’s F-12 (1:1) medium supplemented with 10% FBS, respectively. Neonatal human dermal fibroblasts (nHDFs) were obtained from Invitrogen and grown in DMEM supplemented with 10% FBS.

### Human cytomegalovirus

The HCMV bacterial artificial chromosome (BAC) clone BADrUL131*r*-Y4 that was used to produce infectious virus has been described previously [20]. The HCMV clinical strain TR has been previously described [14]. HCMV virus stocks were prepared by infecting nHDF cells with 0.1 PFU/cell and the cells or culture supernatant was harvested at 10–15 days post-infection. Extracellular virions were concentrated from infected cell supernatants by ultracentrifugation through a 20% (wt/vol) sorbitol cushion at 80,000×g for 1.5 hrs. The virions were then re-suspended in serum-free DMEM and stored at -80°C. For IPs involving HCMV infected cells, nHDFs were seeded in 6-well culture dishes were infected with BADrUL131*r*-Y4 at 1–3 PFU/cell and the cells harvested at 3 days post-infection and solubilized in 1% NP-40.

For IPs involving extracellular virions, virions were produced and purified from 10 day infected nHDFs as described above except that virion pellets were solubilized in 1% NP-40.

### Adenovirus vectors

Construction and production of non-replicating (E1-) adenovirus (Ad) vectors expressing HCMV gB, gH, gL, mutant or tagged versions of HCMV gB have been described previously [18,24,32,33]. Ad vectors expressing HSV-1 gH/gL, gB, and gD, and nectin-1 were constructed as described previously [15,34]. Ad vectors were used to transduce cells at 10–50 PFU/cell. Ad vectors that contained HCMV genes that were regulated by promoters activated by the tetracycline trans activator protein were co-transduced into cells along with the Ad vector, Ad-tet-trans that expresses the tet-trans activator protein [35].

### Antibodies

HCMV specific monoclonal Abs were all kindly provide by Dr. William Britt (University of Alabama, Birmingham) and described previously: anti-gH MAbs 14-4b and AP-86, anti-gB MAb 27–156 [36–38], anti-gM mouse MAb [39] and anti-gB 758 MAb (produced by Scott Koenig, Medimmune Corp.). Rabbit polyclonal anti-peptide sera specific for HCMV gL and UL130 were described previously [21]. Rabbit polyclonal anti-peptide serum specific for gO (TBgO) [23] was kindly provided by Dr. Brent Ryckman (University of Montana, Missoula). HSV-1 antibodies were described previously: Anti gD MAb DL6, [40], anti-HSV-1 gH MAb 53S [41], anti-HSV-1 gB SS10 [42] anti-gD (R45) [43], HSV-1 gH rabbit polyclonal anti-serum R137 [44] and HSV-1 gB rabbit polyclonal antiserum R68 [45]. The polyclonal HCMV gB anti-serum was produced in rabbits at OHSU using the soluble recombinant gB4M [19] according to standard protocols [19,46]. Anti-gB MAbs, 9C1, 13H10, and 15H7 were made at the OHSU monoclonal antibody core facility with mice injected with soluble gB4M. Anti FLAG M2 MAb was from Sigma. The rabbit polyclonal antibody to calnexin (SPA-865) was obtained from Stressgen.

### Immunoprecipitations and western blotting

Ad-transduced or HCMV infected cell monolayers or purified HCMV virus particles were solubilized in Tris-buffered saline (TBS) (50mM Tris, pH 7.4, 150 mM NaCl) containing 1% Nonidet P-40 (NP-40), transferred to microfuge tubes and incubated on ice for 10 min. Non-soluble material was removed by centrifuging lysates for 5 min at 12,000×g in a microcentrifuge. Lysates were incubated in primary antibody for 4 hrs or overnight at 4°C followed by incubation with protein-A agarose for 1 hr at 4°C. Immunocomplexes were collected by centrifugation at 500×g, washed 3 times with TBS containing 1% NP-40 and eluted in sample loading buffer (50mM Tris-pH 6.8, 10% glycerol and 2% SDS) with or without 1% 2-mercaptoethanol. Precipitated proteins were separated using standard SDS-polyacrylamide electrophoresis and then electro-transferred to polyvinylidene fluoride (PVDF) membranes. Electrophoresis of gM was performed using polyacrylamide gels containing 3M urea as previously described [39]. Membranes were incubated in 50 mM Tris-HCl, pH 7.5, 150 mM NaCl, 0.1% Tween-20 (TBST) containing 5% non-fat milk, washed, followed by incubation in TBST with primary antibodies for 1hr or overnight at 4°C. Membranes were washed 3 times for 10 min in TBST and incubated in TBST with horseradish peroxidase-conjugated secondary antibodies for 1h. Lysates of gH/gL expressing ARPE-19 cells were derived from cells transduced with Ad vectors expressing gH/gL as described above. Cells were solubilized in 1% NP-40 buffer and the total protein concentration of the lysate was determined using the Bio-Rad protein assay. Proteins were detected by incubating membranes in chemiluminescent reagent (Perkin Elmer) according to the manufacturer’s instructions and imaged with an Imagequant LAS 4000 system (GE Healthcare). The density of bands on western bots was quantified using ImageJ software or Image studio (Licor).

### Radioactive labeling and immunoprecipitations

Ad-transduced or transfected cell monolayers were washed in PBS and then incubated for 20 min. in starve media consisting of cysteine/methionine free DMEM containing 2% dialyzed FBS and 10 mM HEPES, pH 7.3. The media was then replaced with fresh starve media supplemented with 100μCi/ml ^35^S (Express Protein labeling mix, Perkin Elmer). Cells were labeled for 4 hrs, collected, and solubilized in IP buffer containing 1% NP-40, 1% digitonin, or 1% octyl glucoside. For pulse-chase labeling, Ad-transduced cells in 10 cm plates were removed from the plates with trypsin and washed twice in starve media. Cells from each plate were transferred into two microfuge tubes and incubated in 250 μl starve media for 2 hrs. The media was then exchanged with 250 μl of starve media supplemented with 1mCi/ml ^35^S Express Protein labeling mix. Cells were labeled for 15 min. and then the radioactive cysteine/methionine chased out by addition of DMEM with 10% FBS and 10-fold excess nonradioactive methionine and cysteine (chase media). Cells were then incubated in chase media before being collected at different time-points. Lysates from labeled cells were used for immunoprecipitation and analyzed by SDS-PAGE as described above.

### Construction and expression of mutant gH and gL molecules

Mutant gH and gL molecules were generated by using an overlapping oligonucleotide-directed site-specific strategy with a standard two-step PCR protocol with TR gH and gL gene sequences as templates. Linear sequences encoding 2–5 amino acids were replaced with the same number of alanines. An oligonucleotide was also used to add a FLAG epitope (DYKDDDDK) tag onto the C-terminus of the gH open reading frame. The modified gL and gH gene sequences were cloned into the mammalian expression plasmid pcDNA 3.1+, or pSPORT6 (Invitrogen), respectively, and sequence verified. gH and gL plasmids along with a pSPORT6 plasmid expressing TR gB were used to transfect 293T cells. A total of 2 μg of plasmid DNA was transfected into one well of a 6-cell culture dish of 293T cells using Lipofectamine 2000 (Invitrogen) for 48 hrs then lysed in 1% NP-40 and the cell extracts subjected to IP with an anti-FLAG MAb and IP of gH/gL and gB analyzed by SDS-PAGE.

## Supporting Information

S1 Table Characterization of gH mutants. A complete list of the gH mutations that were generated and tested in IP experiments. Explanation of nomenclature: the gH mutant 140SQQLK, contained the sequence: serine-140, glutamine-141, glutamine-142, leucine-143, and lysine-144 that were all changed to alanine. The mutant forms of gH were fused to a C-terminal FLAG epitope tag. The gH mutant molecules listed in column one were co-expressed with wild type gL, gB, gO, or UL128-131 molecules in 293T cells. The cells were then radiolabeled and gH IP’d with an anti-FLAG MAb. Proteins were separated by SDS-PAGE and analyzed for co-IP of gL (column two), gB (column three), gO (column four) or UL128 (column five). Certain mutant gH molecules were also IP’d with the conformational-specific anti-gH MAb 14-4b (column six). n/a indicates that IPs were not performed
(TIF)

S2 Table Characterization of gL mutants. The mutagenesis strategy and nomenclature for the gL mutant molecules is the same as that described for the gH mutants with exception of the cysteine mutant, C144S in which the cysteine at position 144 was replaced with a serine. The gL mutant molecules listed in column one were co-expressed with wild type gH and gB in 293T cells. The cells were then radiolabelled and proteins IP’d with anti-gH MAb 14-4b. Proteins were separated by SDS-PAGE and analyzed for co-IP of gL with gH (column two) or gB (column three).
(TIF)
