# Supplementary figures and images for: Human Cytomegalovirus gH/gL Forms a Stable Complex with the Fusion Protein gB in Virions
Source: PLoS Pathog. 2016 Apr 15;12(4):e1005564. doi: 10.1371/journal.ppat.1005564 (PMC4833381; doi:10.1371/journal.ppat.1005564)

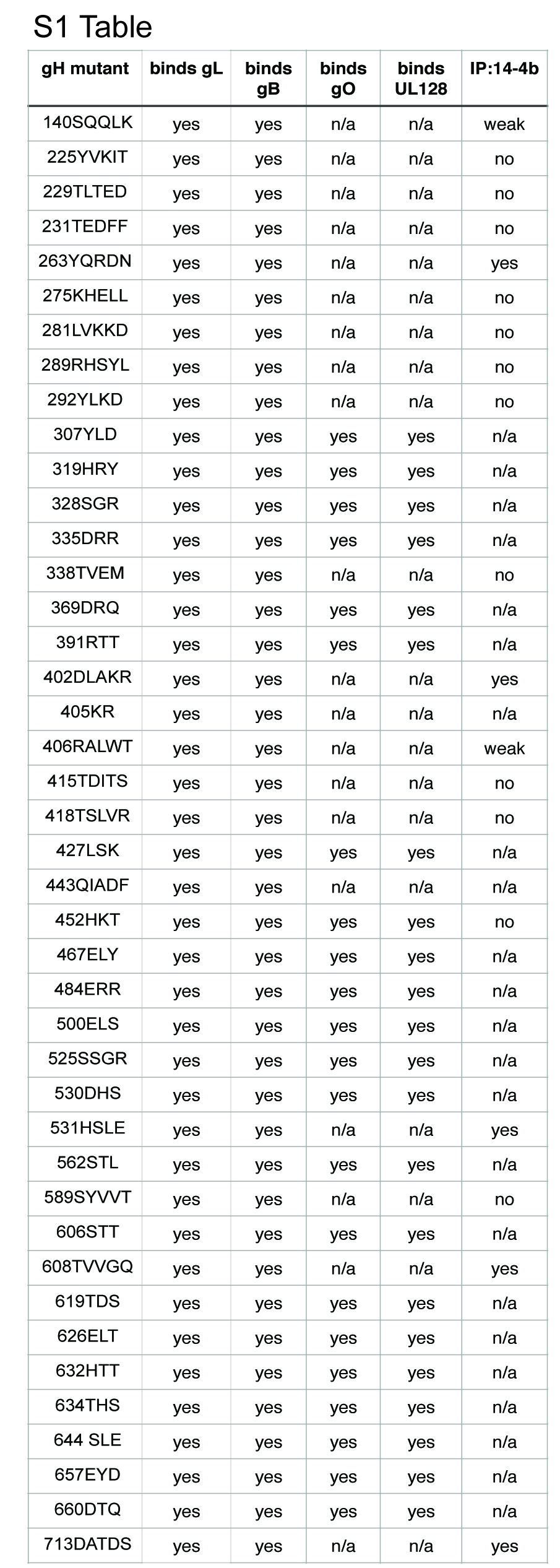

Supplement: S1 Table — A complete list of the gH mutations that were generated and tested in IP experiments. Explanation of nomenclature: the gH mutant 140SQQLK, contained the sequence: serine-140, glutamine-141, glutamine-142, leucine-143, and lysine-144 that were all changed to alanine. The mutant forms of gH were fused to a C-terminal FLAG epitope tag. The gH mutant molecules listed in column one were co-expressed with wild type gL, gB, gO, or UL128-131 molecules in 293T cells. The cells were then radiolabeled and gH IP’d with an anti-FLAG MAb. Proteins were separated by SDS-PAGE and analyzed for co-IP of gL (column two), gB (column three), gO (column four) or UL128 (column five). Certain mutant gH molecules were also IP’d with the conformational-specific anti-gH MAb 14-4b (column six). n/a indicates that IPs were not performed (TIF) [file ppat.1005564.s001.tif]

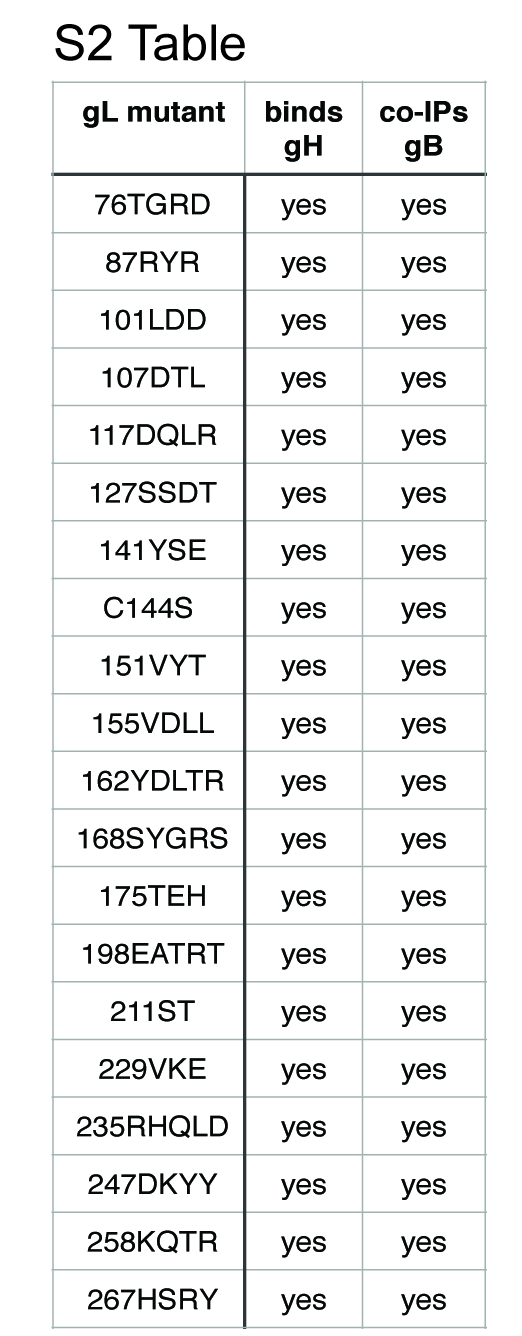

Supplement: S2 Table — The mutagenesis strategy and nomenclature for the gL mutant molecules is the same as that described for the gH mutants with exception of the cysteine mutant, C144S in which the cysteine at position 144 was replaced with a serine. The gL mutant molecules listed in column one were co-expressed with wild type gH and gB in 293T cells. The cells were then radiolabelled and proteins IP’d with anti-gH MAb 14-4b. Proteins were separated by SDS-PAGE and analyzed for co-IP of gL with gH (column two) or gB (column three). (TIF) [file ppat.1005564.s002.tif]
